# Interaction of cold radiofrequency plasma with seeds of beans (*Phaseolus vulgaris*)

**DOI:** 10.1093/jxb/erv206

**Published:** 2015-05-06

**Authors:** Edward Bormashenko, Yekaterina Shapira, Roman Grynyov, Gene Whyman, Yelena Bormashenko, Elyashiv Drori

**Affiliations:** ^1^Ariel University, Physics Faculty, POB 3, 40700, Ariel, Israel; ^2^Ariel University, Chemical Engineering and Biotechnology Faculty, POB 3, 40700, Ariel, Israel; ^3^Agriculture Research Department, The Samaria and Jordan Rift Regional R&D Center, Science Park, 40700, Ariel, Israel

**Keywords:** Beans, contact angle, germination, plasma treatment, water imbibition, wetting properties.

## Abstract

The impact of cold radiofrequency air plasma on the wetting properties and water imbibition of beans (*Phaseolus vulgaris*) was studied. The influence of plasma on wetting of a cotyledon and seed coat (testa) was elucidated. It was established that cold plasma treatment leads to hydrophilization of the cotyledon and tissues constituting the testa when they are separately exposed to plasma. By contrast, when the entire bean is exposed to plasma treatment, only the external surface of the bean is hydrophilized by the cold plasma. Water imbibition by plasma-treated beans was studied. Plasma treatment markedly accelerates the water absorption. The crucial role of a micropyle in the process of water imbibition was established. It was established that the final percentage of germination was almost the same in the cases of plasma-treated, untreated, and vacuum-pumped samples. However, the speed of germination was markedly higher for the plasma-treated samples. The influence of the vacuum pumping involved in the cold plasma treatment on the germination was also clarified.

## Highlight

The impact of cold plasma on the wetting, water absorption, and germination of beans (*Phaseolus vulgaris*) is reported. Plasma treatment accelerated the water absorption and germination of seeds.

## Introduction

The interaction of various kinds of radiation with plants has been exposed to intensive research (Teramura, 1983; Cen and Bornman, 1990; Jansen *et al.*, 1998; Friesen *et al.*, 2014, Guajardo-Flores *et al.*, 2014). Generally, the researchers focused on the interaction of UV radiation with plants (Cen and Bornman, 1990; Guajardo-Flores *et al.*, 2014). One kind of interaction remains practically uninvestigated, namely the interaction of cold radiofrequency plasma with plants. The first reports in this field appeared only in the last two decades. It seems that the first systematic study of the influence exerted by plasma on seeds was carried out by Volin *et al.* (2000) who exposed seeds of radish (*Raphanus sativus*) and two pea cultivars (*Pisum sativum* cv. ‘Little Marvel’, *P. sativum* cv. ‘Alaska’) to CF_4_ and octadecafluorodecalin plasma, and reported a significant delay in germination compared with the untreated controls. Since then, researchers have concentrated on the following main trends of investigation: (i) decontamination of seeds by plasma, (ii) breaking of dormancy with plasma, (iii) the impact of plasma treatment (PT) on germination, and (iv) the impact of PT on root generation (sprouting).

Decontamination and inactivation of pathogenic microorganisms of seeds have been communicated recently by various groups (Selcuk *et al.*, 2008; Filatova *et al.*, 2009; Schnabel *et al.*, 2012). Several groups reported the impact of PT on germination, sprouting, and dormancy breaking of seeds. The experimental data in these fields are scanty and controversial. Sera *et al.* (2010) investigated the influence of PT on wheat and oat germination. The authors reported that PT did not affect the germination of oat seeds, but they did note accelerated root generation in plants grown from plasma-treated seeds (Sera *et al.*, 2010). The same group also showed that PT did change seed germination in Lamb’s Quarters seeds (Sera *et al.*, 2009). A stimulating effect of cold plasma on both the germination and sprouting of tomato seeds (*Lycopersicon esculentum* L. Mill. cv. ‘Zhongshu No. 6’) has been reported by Meiqiang *et al.* (2005). Similar results were reported for *Pauwlonia tomentosa* seeds (Živković *et al.*, 2004). Kitazaki *et al.* (2012) studied the growth enhancement of radish sprouts (*Raphanus sativus* L.) induced by low pressure O_2_ radiofrequency plasma irradiation. The experimental results revealed that oxygen-related radicals strongly enhance growth, whereas ions and photons do not (Kitazaki *et al.*, 2012). The positive effect of cold helium plasma treatment on seed germination, growth, and yield was reported recently for wheat (Jiafeng *et al.*, 2014). Treatment of spinach seeds by a magnetized arc plasma increased the germination rate by 137% (Shao *et al.*, 2013). It has been demonstrated that cold atmospheric plasma treatment had little effect on the final germination percentage of radish seeds, but it influenced their early growth (Mihai *et al.*, 2014). Generally, the researchers agree that cold plasma treatment is an economical and pollution-free method to improve seed performance and crop yield. It plays essential roles in a broad spectrum of developmental and physiological processes in plants, including reducing the bacterial bearing rate of seeds, changing seed coat structures, increasing the wettability and permeability of seed coats, and stimulating seed germination and seedling growth (Selcuk *et al.*, 2008, Jiafeng *et al.*, 2014; Ling *et al.* 2014).

Our group recently studied the influence of cold air plasma on lentils (*Lens culinaris*), beans (*Phaseolus vulgaris*), and wheat grains (*Triticum* sp. *C9*) (Bormashenko *et al.*, 2012). It was established that plasma treatment dramatically influenced the wettability of the seeds, resulting in a significant decrease of the apparent contact angle (de Gennes et al., 2003; Erbil, 2006). The change in wettability is followed by a consequent change in the water imbibition of the seeds. It was also reported that cold PT markedly modifies the wettability of various biological tissues, including lycopodium particles and keratin (Bormashenko and Grynyov, 2012a, *b*). The impact of cold plasma on the wettability of keratin has also been reported by Molina *et al.* (2003).

Hydrophilization of biological tissues by cold plasma resembles the similar effect observed and deeply researched in synthetic polymers (Yasuda, 1976; Strobel *et al.*, 1994; France and Short, 1998; Stoffels *et al.*, 2008).The plasma treatment of synthetic polymers creates a complex mixture of surface functionalities which influence surface physical and chemical properties and results in a dramatic change in the wetting behaviour of the surface (France and Short, 1997; Kaminska *et al.*, 2002). Not only the chemical structure but also the roughness of the surface is affected by the plasma treatment; this also could change the wettability of the surface (Lommatzsch *et al.*, 2007). PT usually strengthens the hydrophilicity of treated synthetic polymer surfaces. However, the surface hydrophilicity created by plasma treatment is often lost over time (Morra *et al.*, 1990). This effect of decreasing hydrophilicity is called ‘hydrophobic recovery’ (Morra *et al.*, 1990; Occhiello *et al.*, 1992; Kaminska *et al.*, 2002; Pascual *et al.*, 2008; Mortazavi *et al.*, 2012). By contrast, in our recent research, hydrophobic recovery was not observed in plasma-treated seeds (Bormashenko *et al.*, 2012). It should be emphasized that cold plasma treatment only influences the nano-scaled external layer of a tissue (France and Short, 1997; Kaminska *et al.*, 2002; Mortazavi and Nosonovsky, 2012; Bormashenko *et al.*, 2013). This fact may be crucial for the biological applications of cold plasma treatment.

There are still many open issues with regard to the mechanisms of plasma action on synthetic polymers. Obviously, the processes of interaction with biological objects such as seeds are even more complicated. In this paper, the focus is on the modification of wetting properties by plasma, studied for a variety of interfaces constituting beans (exotesta, mesotesta, and cotyledon), change in water imbibition due to the plasma treatment, and also on the effect of plasma treatment on the germination of beans. It is noteworthy that beans are amenable for the goniometric study of the effect of plasma on their wettability represented by an apparent contact angle due to their large surface area. Changes in the water imbibition pathways due to the plasma treatment were also tracked, as well as the effect of plasma treatment on the germination kinetics of beans. Our research demonstrates that the speed of germination may be affected by the cold plasma treatment.

## Materials and methods

### Plasma treatment of beans

**Fig. 1.**
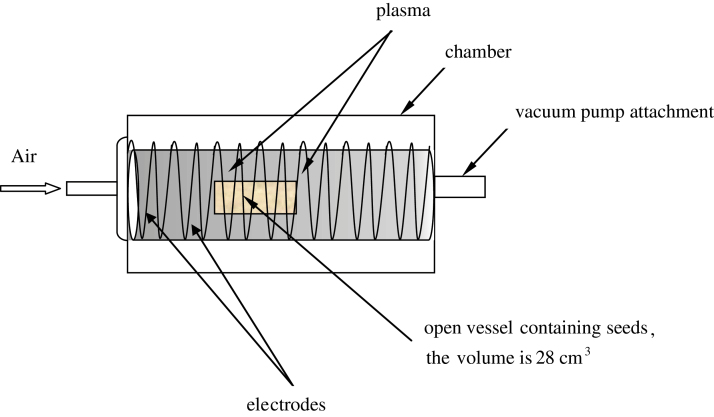
Plasma unit used for treatment of seeds.

PT was carried out with the plasma unit EQ-PDC-326, manufactured by MTI Co, USA. Beans (*Phaseolus vulgaris*) were exposed to an inductive air plasma discharge under the following parameters: the plasma frequency was on the order of 10 MHz, the power was 20W, the pressure was 6.7×10^–2^ Pa; the volume of the discharge chamber was 840cm^3^. The time span of irradiation was 2min. The scheme of the experimental unit used for plasma treatment of the seeds is depicted in Fig. 1. Under plasma treatment, the beans were exposed to low vacuum; hence it was necessary to study separately the impact of a vacuum on the properties of beans.

### Study of the impact of vacuum pumping on the properties of beans

Beans were exposed to a low vacuum with the pressure of 6.7×10^–2^ Pa for 3min. Ten beans were weighed before and immediately after vacuum pumping. The weight was taken with a MRCASB-220-C2 five place analytical balance. Thus, any weight loss of beans occurring under vacuum pumping was established. Afterwards, the vacuumed beans were exposed to ambient conditions: the average temperature was 22.3 °C; the relative humidity was 31–33%. The weight of the beans was measured continuously over 5min. Thus, the kinetics of water adsorption by the beans was established.

### SEM imaging of beans

Irradiated and non-irradiated beans and tissues (a cotyledon and seed coat) were imaged by high resolution SEM (JSM-6510 LV).

### Trinocular imaging of beans

Irradiated and non-irradiated beans and tissues constituting the seed were imaged by the trinocular microscope (Model SZM-2) with a USB digital camera (OPTIKAM PRO3) supplied by Optika Microscopes Italy.

### Study of the wetting properties of tissues

The wetting properties of tissues were established using a Ramé–Hart goniometer (model 500). Ten measurements were taken to calculate the mean apparent contact angles (de Gennes *et al.*, 2003; Erbil, 2006) for all kinds of studied tissues.

### Study of water imbibition

For the study of the time dependence of water absorption (imbibition) by irradiated and non-irradiated beans, 10 seeds were placed on humid blotting paper at ambient conditions; the temperature was 24 °C. Beans were weighed every hour with an MRC ASB-220-C2 analytical balance. The relative water imbibition (absorption) was defined as: $\frac{\Delta m(t)}{m_{0}}100\%=\frac{m(t)-m_{0}}{m_{0}}100\%$, where *m*_0_ is the total initial mass of seeds, and *m*(*t*) is the running total mass of seeds. A series of six experiments was performed for untreated, vacuumed (pumped), and plasma-treated beans.

### Study of the role of a micropyle in water imbibition

For the study of the role of the micropyle in water imbibition, experiments with open and sealed micropyles were performed. The micropyle was sealed with thermosetting glue (UHU 41686 Instant Super Glue).

### Direct visualization of the water penetration

Untreated and plasma-treated beans were immersed in water coloured with Bromophenol Blue (0.33 wt.%) for 5h. Bromophenol blue (3′,3″,5′,5″-tetrabromophenol sulphonphthalein) was supplied by Sigma Aldrich, Israel.

### Study of impact of the PT on beans’ germination

For the study of the impact of PT on germination, 30 seeds of irradiated, vacuum-pumped, and non-radiated beans were placed on humid blotting paper for about 80h at constant conditions provided by a growth chamber (Model PGI-500H, supplied by MRC Ltd, Israel); the temperature was kept at 21 °C; the process included 12h of darkness and 12h of light per day. Every 4–8h the numbers of emerged seedlings were counted. The percentage of germination was calculated after counting seedlings on the fourth day. Two sets of 180 seeds were taken for vacuum and plasma treatments, respectively; the control group also included 180 seeds. All experiments were repeated three times.

The results of the experiments were processed by JMP software (Statistical Discovery, Version 7.0.2).

## Results and discussion

### Impact of vacuum pumping on the properties of beans

**Fig. 2.**
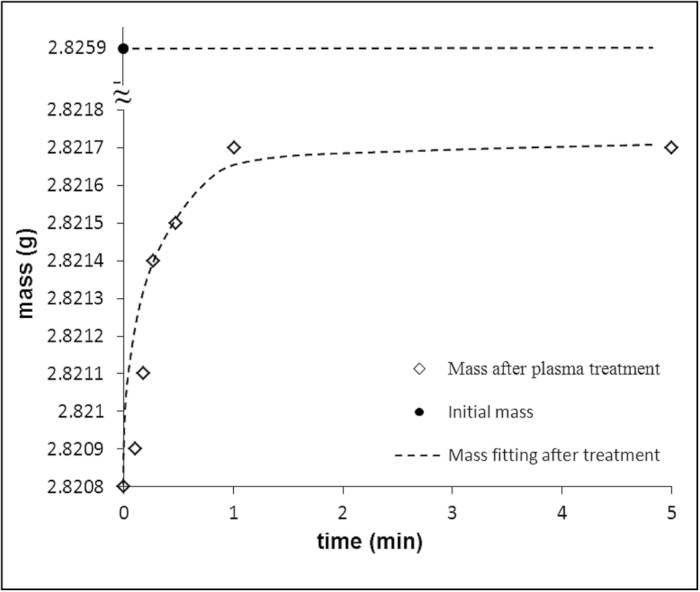
Weight loss of beans (*n*=10) due to the pumping followed by the increase due to the water adsorption. The size of diamonds denoting experimental points indicates the experimental scattering of the results.

When beans are treated with cold plasma, they are necessarily exposed to a vacuum. They also lose weight when pumped and it is possible that their germination rate would also be changed (the germination of beans will be discussed later). The weight loss and water adsorption of pumped beans were established (see Materials and methods: study of the impact of vacuum pumping on the properties of beans). The average weight loss of pumped beans was established as 0.11% compared with the starting weight. Pumped beans partially restored their weight when exposed to a humid atmosphere (for details see Materials and methods: study of the impact of vacuum pumping on the properties of beans). It is plausible to suggest that this restoration is governed by surface events, i.e. water adsorption to the beans’ surface. The dynamics of water adsorption by pumped beans is illustrated in Fig. 2. First of all, it should be stressed that pumped beans did not restore their initial weight. This means that pumping removes water not only from the surface of beans but also from the beans’ bulk. The time dependence of water adsorption of pumped beans is well fitted by Equation 1.

$$m(t)=m_{0}+\Delta m_{ad}[1-\exp(-\frac{t}{\tau })]$$ (1)

where *m*(*t*) is the running mass, $m_{0}$ is the initial mass established immediately after pumping, *t* is time, *τ* is the characteristic time of adsorption and $\Delta m_{ad}$ is the resulting mass of adsorbed water. The average characteristic time of adsorption *τ* was established by fitting of experimental data with Equation 1 as 0.38±0.07min.

### Impact of the plasma treatment on the wettability of different biological tissues constituting seeds

**Fig. 3.**
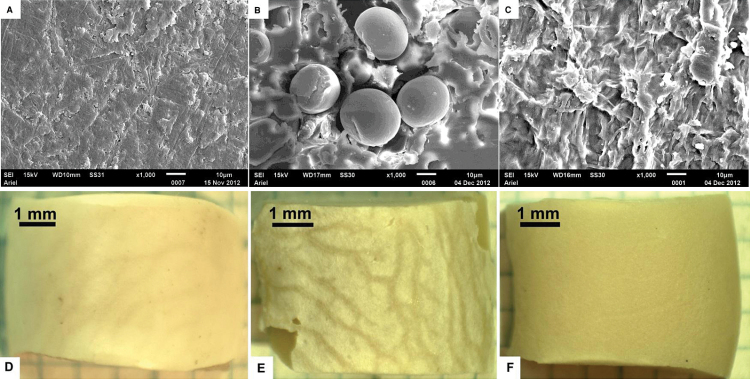
SEM images of: (A) the exotesta, (B) the mesotesta, (C) a cotyledon surface, the scale bars=10 μm. Trinocular images of: (D) the exotesta; (E) the mesotesta; (F) a cotyledon surface.

It has already been reported that cold radiofrequency PT dramatically changed the wettability of the beans’ seed coat (Bormashenko *et al.*, 2012). A similar change in the wetting of soybeans exposed to cold plasma was reported recently (Ling *et al.*, 2014). PT treatment markedly hydrophilized and irreversibly changed the wetting regime of the external side of the seed coat. It was also important to establish the impact exerted by PT on the wettability of other tissues constituting the seed: the testa, including the exotesta and mesotesta, and the cotyledon epidermis (Aniszewski *et al.*, 2006). For this purpose, two series of experiments were carried out. In the first series, intact beans were exposed to the plasma treatment, as described in the Materials and methods: plasma treatment of beans, and, afterwards, the testa and exotesta were separated from the cotyledon using a scalpel and tweezers. The SEM and trinocular images of the inner (mesotesta)/outer (exotesta) sides of the seed coat and cotyledon surface are presented in Fig. 3A–F (for the experimental details see the Materials and methods: SEM and trinocular imaging of beans).

In the second series, the testa was separated from the cotyledon, and they were individually exposed to the PT, as described in the Materials and methods: Plasma treatment of bean*s*. The wettability of irradiated and non-irradiated tissues was established by the measurement of apparent contact angles (APCAs) as described in the Materials and methods: Study of the wetting properties of tissues. The results of the measurements are summarized in Tables 1 and 2, where $\theta_{EXO}^{0}$ is the APCA of the exotesta before PT, $\theta_{\begin{array}{l} EXO \end{array}}^{PT}$ is the APCA of the exotesta after PT, $\theta_{MESO}^{0}$ is the APCA of the mesotesta before PT, $\theta_{MESO}^{PT}$ is the APCA of the mesotesta after PT, $\theta_{COT}^{0}$ is the APCA of the cotyledon before PT, and $\theta_{COT}^{PT}$ is the APCA of the cotyledon after PT.

**Table 1.** Apparent contact angles (in degrees) as established for different tissues for entire beans exposed to PT (subscripts: EXO, exotesta; MESO, mesotesta; COT, cotyledon; superscripts: 0, untreated beans; PT, plasma-treated beans)

$\theta_{EXO}^{0}$	$\theta_{\begin{array}{l} EXO \end{array}}^{PT}$	$\theta_{MESO}^{0}$	$\theta_{MESO}^{PT}$	$\theta_{COT}^{0}$	$\theta_{COT}^{PT}$
109±1	40±1	132±1	131±2	101±2	100±2

**Table 2.** Apparent contact angles (in degrees) as established for different tissues for seed coatings and cotyledons exposed separately to plasma treatment

$\theta_{EXO}^{0}$	$\theta_{\begin{array}{l} EXO \end{array}}^{PT}$	$\theta_{MESO}^{0}$	$\theta_{MESO}^{PT}$	$\theta_{COT}^{0}$	$\theta_{COT}^{PT}$
109±1	40±1	132±1	0	101±2	72±1

**Fig. 4.**
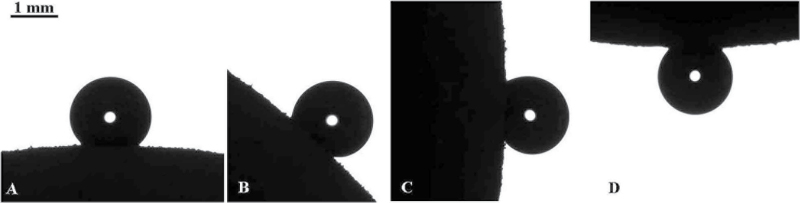
The ‘rose petal effect’ observed on the mesotesta of bean seeds. High apparent contact angles are attended by a high adhesive wetting state. The droplet is attached to the surface even in the pendant position.

First of all, it should be stressed that the mesotesta demonstrated an apparent contact angle as high as 132°, as depicted in Fig. 4A, which is close to the APCA inherent to superhydrophobic surfaces (Barthlot *et al.*, 1997; Nosonovsky, 2007; Bhushan *et al.*, 2010; Bormashenko *et al.*, 2013). The high values of APCA are due to the multiscale roughness observed on the mesotesta, shown in Fig. 3B (Nosonovsky, 2007). However, the mesotesta does not demonstrate a true superhydrophobicity, but rather the so-called ‘rose petal effect’, where high values of APCA are attended by a high adhesion wetting regime, illustrated in Fig. 4B–D. Indeed, a water droplet remained connected to the mesotesta even in the ‘pendant’ position, as shown in Fig. 4B.

It should be emphasized that the mesotesta kept its pronounced hydrophobicity when the entire bean was exposed to PT. The APCA did not change, as seen from the data supplied in Table 1. The cold plasma comprises a variety of species, including atoms, ions, electrons, and photons (Lieberman *et al.*, 2005). However, the changes in wettability of organic tissues produced by cold plasma are mainly ascribed to the collisions of ions with moieties constituting a tissue. Indeed, the energy transfer related to collisions of electrons with a tissue is negligible, and the energy of photons is deficient for the essential changes in the wettability of a tissue (Lieberman *et al.*, 2005). Thus, the absence of changes in the wettability of the mesotesta leads to the suggestion that ions lost their energy when penetrating through the coat, and these ions are incapable of modifying the wetting regime of the surface.

It is also seen from data, presented in Table 1, that the wetting properties of the cotyledon were similarly not changed by plasma when entire beans were treated. Thus, the absence of changes in the wettability of the cotyledon strengthens the hypothesis that the plasma species ‘attenuated’ by the coat do not have sufficient energy for the modification of the wettability of the internal sides of the biological surfaces studied in our research. By contrast, cold plasma markedly hydrophilized all the biological surfaces studied when they were separately exposed to PT, as seen from the data presented in Table 2. A jump from pronounced hydrophobicity to complete wetting was observed for the strongly hydrophobic mesotesta. Changes in APCA as high as 69° and 28° were observed for the exotesta and cotyledon. These findings coincide with those reported in our previous work (Bormashenko *et al.*, 2012). It is concluded that PT modifies the wetting of various tissues constituting beans, when the access of ions to the tissues is not restricted.

### Study of the water absorption by untreated and plasma-treated beans

As shown in the previous section, PT modifies the wetting regime of tissues and promotes their hydrophilization. This effect leads to increased water absorption (imbibition) of plasma-treated seeds, as demonstrated in our recent paper (Bormashenko *et al.*, 2012). We now report more detailed study of water imbibition by plasma-treated beans, in which the pathways of water imbibition were investigated. The water imbibition occurring in plasma-treated beans is a complicated process, influenced by a number of factors. (i) It was demonstrated in ‘Impact of vacuum pumping on the properties of beans*’* that vacuum pumping removes water not only from the surface of beans, but also from their bulk. Thus, it is reasonable to suggest that vacuum pumping will also influence the process of water imbibition. (ii) The seed coating inherent for beans is not intact; it contains a small opening called a ‘micropyle’ (Bewley and Black, 1994). The crucial role of the micropyle in water imbibition is widely acknowledged (Hagon, 1970; Rolston, 1978; Gama-Arachchige *et al.*, 2013).

**Fig. 5.**
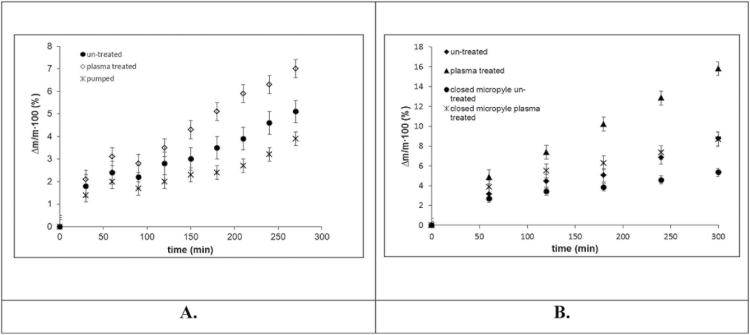
Water absorption by beans. (A) Comparative study of water imbibition by untreated, pumped without plasma, and plasma-treated beans. (B) Comparative study of water imbibition by non-treated beans with an open micropyle, plasma-treated beans with an open micropyle, untreated beans with a closed micropyle, plasma-treated beans with a closed micropyle.

In order to separate the impact of factors influencing water imbibition, two series of experiments were performed. In the first series, water imbibition of untreated, vacuum-pumped (without plasma), and plasma-treated beans was studied, as described in ‘Study of water imbibition’. In these experiments the micropyle was open. The results of these experiments are depicted in Fig. 5A. It was seen that PT markedly promotes water imbibition. It is also recognized from data supplied in Fig. 5A that vacuum pumping retards water absorption. This observation calls for future investigation.

The second series of experiments was intended to reveal the precise role of the micropyle in water imbibition. For this purpose, the micropyle was sealed precisely with a small droplet of glue (for details, see the Material and methods: study of the role of a micropyle in water imbibition). Thus water imbibition by four kinds of beans was studied, namely: untreated beans with an open micropyle, plasma-treated beans with an open micropyle, untreated beans with a sealed micropyle, and plasma-treated beans with a sealed micropyle. Their water imbibition was studied as described in the Materials and methods: study of water imbibition; the results are depicted in Fig. 5B. It is distinctly seen that the fastest water imbibition was observed for plasma-treated beans with an open micropyle. The slowest water absorption was registered for untreated beans with a sealed micropyle. Plasma treated beans with a sealed micropyle demonstrated a speed of water imbibition higher than that of untreated beans with a sealed micropyle, but remarkably slower than that of plasma-treated beans with open (unsealed) micropyles. Thus, the strengthening impact of plasma treatment on water imbibition through the beans’ coat, as well as the essential role of the micropyle, were both established.

**Fig. 6.**
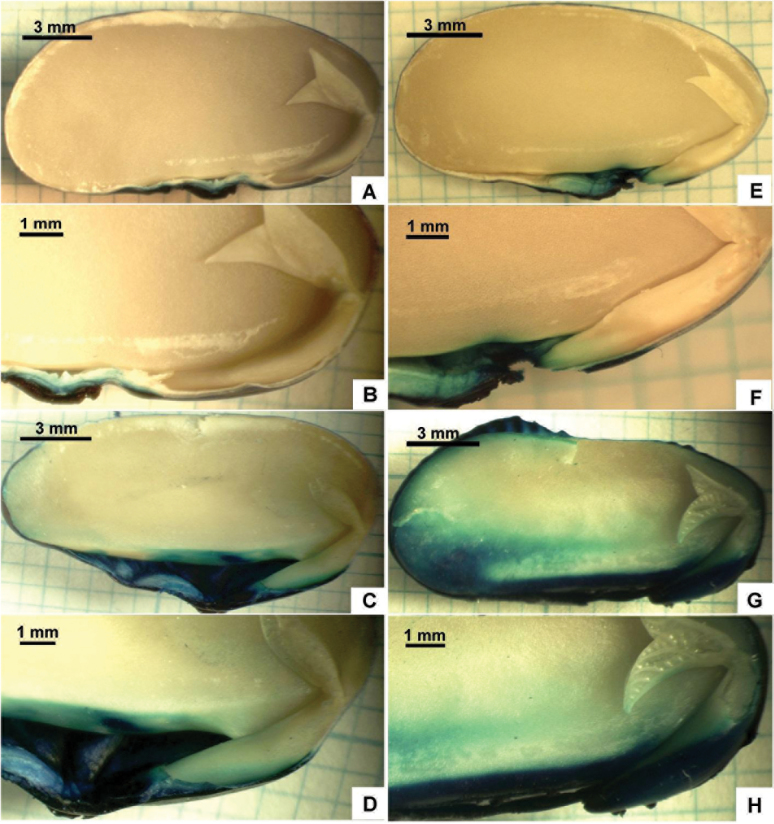
Trinocular images of water penetration in plasma treated (E–H) and non-treated (A–D) beans. (A, B) untreated bean after 2h in coloured water; (C, D) untreated bean after 5h in coloured water; (E, F) plasma-treated bean after 2h in coloured water; (G, H) plasma-treated bean after 5h in coloured water. Water was coloured with Bromophenol Blue. The micropyle is open.

Qualitative experiments were also performed which shed light on the kinetics of water imbibition. Untreated and plasma-treated beans were immersed in water coloured with Bromophenol Blue (see the Materials and methods: direct visualization of water penetration), which visualized the process of water penetration into the beans. Immersed beans were cut every hour, and a distinct boundary was observed, separating areas wet by coloured water and non-wet ones, as shown in Fig. 6A–H, where: (A, B) is the untreated bean after 2h in coloured water; (C, D) is the untreated bean after 5h in coloured water; (E, F) is the plasma-treated bean after 2h in coloured water; (G, H) is the plasma-treated bean after 5h in coloured water. It was clearly seen that more rapid penetration occurs in plasma-treated beans. The micropyles in these experiments were open. It can also be recognized from Fig. 6A–H that water penetrates into beans from the side of a micropyle. This further demonstrates the key role of a micropyle in the process of water imbibition (Rolston, 1978; Gama-Arachchige *et al.*, 2013).

### The impact of PT on beans’ germination

**Fig. 7.**
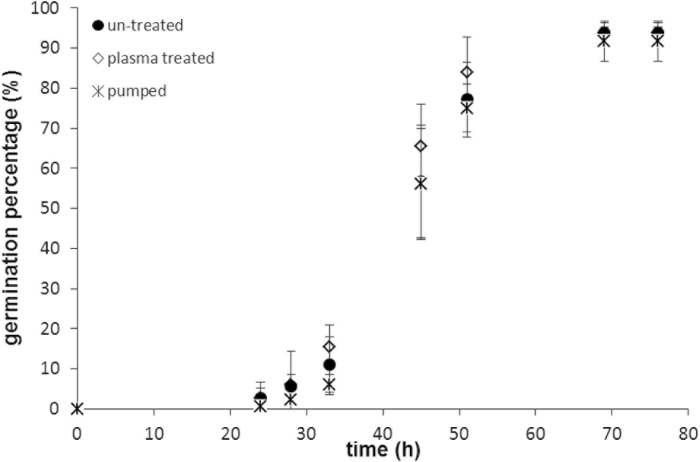
Comparative study of the germination process observed in untreated, vacuum-pumped, and plasma-treated beans.

As shown in the previous sections, PT leads to increased water absorption (imbibition) of seeds. It was also important to verify the impact of increased water absorption on the germination process of seeds. For this purpose, three groups of bean seeds were used: one group of seeds was treated by cold plasma, as described in the Materials and methods: Plasma treatment of beans, the second group was treated by vacuum pumping, and the third group of seeds was untreated. No significant difference was observed in viability (the percentage of germination); all three groups of seeds demonstrated the same viability rates, which were about 92–94%, as depicted in Fig. 7.

**Fig. 8.**
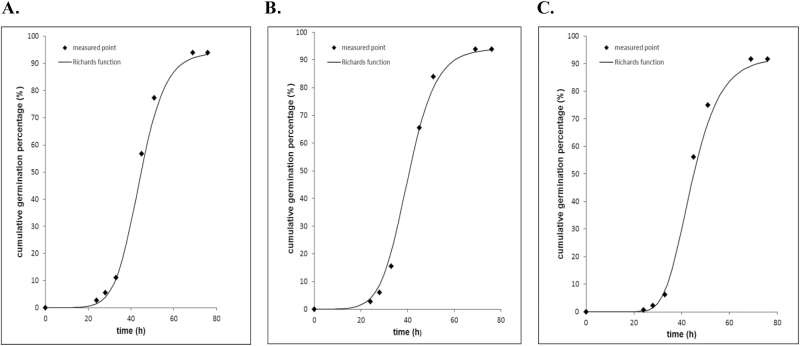
Germination curves calculated using the Richards fitting function. (A) Untreated beans, (B) plasma-treated beans, (C) vacuum-pumped beans.

In order to derive the data describing the kinetics of germination, Richards’curves were fitted to a number of experiments (Richards, 1959; Hara, 1999; Sera *et al.*, 2009). Fitting of experimental data by Richards’ curves is depicted in Fig. 8A–C.). The Richards’ differential equation, originally developed for growth modelling, gives rise to the Richards’ curve, which is an extension of the logistic or sigmoid functions, resulting in *S*-shaped curves describing the kinetics of germination. The Richards’ function *Y*_t_, possessing a variable point of inflection, was calculated according to Equation 2:

Yt=a[1+b×d×exp(−c×t))]1d (2)

where *Y*_t_ is the germination percentage, *a*, *b*, *c*, and *d* are the fitting parameters, and *t* is the time.

Fitting of experimental data by Equation 2 supplied the best values of the fitting parameters, summarized in Table 3, in which *Me* (median) denotes the time of 50% germination and characterizes the rate of this process. The quartile deviation of germination time *Qu* describes the deviation range of the Richards’ curve relative to *Me*, and *Sk* (skewness) represents the asymmetry of the Richards’ curve relative to the inflection point (mode). For calculation of these quantities, the useful formulae developed by Hara (1999) were implemented.

**Table 3.** The population parameters *Vi* (viability), *Me* (median germination time), *Qu* (dispersion), and *Sk* (skewness) of the Richards equation for the germination of beans

Treatment	*Vi* (%)	*Me* (h)	*Qu* (h)	*Sk* (%)
Non-treated	94±1	44±0.001	6.5±0.001	0.17±0.002
Plasma-treated	94±1	40±0.001	6.5±0.001	0.17±0.002
Pumped	92±1	44±0.001	6.5±0.001	0.4±0.002

As seen from Table 3, the final percentage of germination is almost the same in the cases of plasma-treated, untreated, and vacuum pumped samples. The value of *Me*, however, is distinctly lower (by *c*. 4h) in the case of plasma-treated samples. Germination is accelerated as *Me* decreases (Hara, 1999). This means that, although the final germination percentage is the same, the speed of germination is higher for the plasma-treated samples. The value of *Me* was the same for untreated and vacuum-pumped groups. Thus we deduce that vacuum pumping does not influence the rate of germination.

No difference in the parameter *Qu* was established among all groups of seeds (untreated, plasma-treated, and vacuum-pumped). The value of *Sk*=0.17 was the same in the plasma-treated and control groups, whereas it was much higher (*Sk*=0.40) for the vacuum-pumped samples.

## Conclusions

The influence exerted by cold radiofrequency plasma treatment on the wetting properties and germination of beans (*Phaseolus vulgaris*) was investigated. A comparative study of wettability and germination was performed for plasma-treated, vacuum pumped, and untreated seeds. It was established that the cold plasma treatment markedly hydrophilized the external (exotesta) surface of the seed coat, whereas the mesotesta and cotyledon kept their pronounced hydrophobicity when the entire bean was exposed to plasma treatment. It is concluded that plasma ions lose their energy when penetrating through the coat, and these ions are incapable of modifying the wetting regime of the biological surface. Water imbibition into vacuum-pumped and plasma-treated beans was studied. It has been demonstrated that plasma treatment markedly increased the water imbibition of seeds through the testa, independent of the micropyle effect. The key role of a micropyle in the process of imbibition was shown.

The impact of vacuum pumping on the properties of beans, which is inevitable under cold PT, was studied. The kinetics of water adsorption of pumped beans is well fitted by an exponential function. The average characteristic time of adsorption *τ* was established as 0.38±0.07min.

The impact of PT on the germination of beans turned out to be ambiguous. The kinetics of germination was fitted by the Richards curve. It was established that the final percentage of germination is almost the same in the cases of plasma treated, untreated, and vacuum-pumped samples. However, the speed of germination was markedly higher for the plasma-treated samples. It is not known in detail the mechanisms of interaction of cold plasma with the biological tissues constituting seeds; thus detailed microscopic research clarifying this interaction is required.
